# The Real Incidence of Extracapsular (Satellite) Cysts of Liver Echinococcus

**DOI:** 10.1155/1999/62751

**Published:** 1999-07

**Authors:** Dionysios Voros, Angelos Kalovidouris, Athanasios Gouliamos, Lambros Vlachos, Nickolaos Danias, John Papadimitriou

**Affiliations:** 1 2nd Department of Surgery School of Medicine University of Athens Greece; 2 Dept. of Radiology School of Medicine University of Athens Greece; 3 18 Kifissias Ave. Athens 115 26 Greece

## Abstract

*Background:*  The presence of extracapsular (Satellite) cysts in liver echinococcus granulosus is known for many years. In one of our previous studies of radiological (CT) material they were found to be present in 16% of cases.

*Methods:* In the present study the operative findings, in cases of total capsectomy (cystopericystectomy) or partial lobectomy are presented.

*Results:* The real incidence of these cysts in operative specimens was as high as 29,5%. They were present in 15 out of 51 totally excised cysts.

*Conclusions:* We conclude that satellite cysts are present more often than they are radiologically detected. As they can be incriminated as a cause of recurrence of the disease they must be excised en block with the main parasitic cysts, by means of more radical procedures such as cystopericystectomy or partial hepatectomy, whenever it is feasible.


HPB Surgery, 1999, Vol. 11, pp. 249-252
Reprints available directly from the publisher
Photocopying permitted by license only

© 1999 OPA (Overseas Publishers Association) N.V.
Published by license under
the Harwood Academic Publishers imprint,
part of The Gordon and Breach Publishing Group.
Printed in Malaysia.

*Clinical Series*

### The Real Incidence of Extracapsular (Satellite) Cysts of Liver Echinococcus

DIONYSIOS VOROS[a,*], ANGELOS KALOVIDOURIS[b], ATHANASIOS GOULIAMOS[b], LAMBROS VLACHOS[b], NICKOLAOS DANIAS[a] and JOHN PAPADIMITRIOU[a]

[a] *2nd Department of Surgery;* [b] *Dept. of Radiology, School of Medicine, University of Athens, Greece*

*(Received 5 November 1997; In final form 7 November 1998)*

***Background:*** **The presence of extracapsular (Satellite) cysts in liver echinococcus granulosus is known for many years. In one of our previous studies of radiological (CT) material they were found to be present in 16% of cases.**
***Methods:*** **In the present study the operative findings, in cases of total capsectomy (cystopericystectomy) or partial lobectomy are presented.**
***Results*****: The real incidence of these cysts in operative specimens was as high as 29,5%. They were present in 15 out of 51 totally excised cysts.**
***Conclusions:*** **We conclude that satellite cysts are present more often than they are radiologically detected. As they can be incriminated as a cause of recurrence of the disease they must be excised en block with the main parasitic cysts, by means of more radical procedures such as cystopericystectomy or partial hepatectomy, whenever it is feasible.**

*Keywords:* Hydatid or echinococcal cyst of the liver, extracapsular or satellite echinococcal cysts, cystopericystectomy (for hydatid disease), capsectomy total (for hydatid disease)

### INTRODUCTION

The management of hydatidosis continues to be essentially surgical. Various drugs such as Ambedoze, Benzimidazole *etc.* have not given satisfactory results as a primary treatment, and it is widely accepted that they are useful as an adjuvant therapy [1]. Total capsectomy (cystopericystectomy) and partial (non typical) hepatectomy for smaller cysts had been performed for many years as radical operative procedures in liver hydatidosis; the parasite is removed en block without intraoperative spilage, and moreover, the problems of the residual cavity are solved by these methods. These procedures were considered to be of limited applicability because they were thought to lead to increased morbidity and serious complications. However in recent years, especially with the progress in liver surgery, they have emerged to the first line as radical and effective methods [2-5]. They assume recently special significance from the fact that in these procedures, the extracapsular (satellite) cysts, studied in the light of computerized and magnetic tomography, are also removed [6,7]. In the present study the operative

*Address for correspondence: 18 Kifissias Ave., 115 26 Athens-Greece. Tel.: (01) 77 06 152, Fax: (01) 77 85 780.

findings regarding the extracapsular cysts are presented and evaluated.

### METHODS

We studied retrospectively 50 patients with liver echinococcus granulosus scheduled for total capsectomy in our Department during the last five years. Total capsectomy implies the removal of all the parasitic cyst wall without opening the cyst in contrast to extended capsectomy where most of the wall is removed to diminish the remaining cavity and simple or partial capsectomy where unroofing and evacuation of the main cyst is performed.

Coexisting cysts localized in other organs, outside the liver, such as lungs, kidneys, spleen, ovaries, were treated surgically with the aim of radical excision.

All hydatid cysts removed were inspected for the presence of small extracapsular cysts and the operative findings were compared to the preoperative radiological findings.

### RESULTS

All 50 patients were preoperatively investigated by computed tomography (Fig. 1), sixteen had magnetic resonance imaging (MRI) and five patients had angiographic studies. Table I summarizes the location of hydatid cysts according to the preoperative radiological examinations.

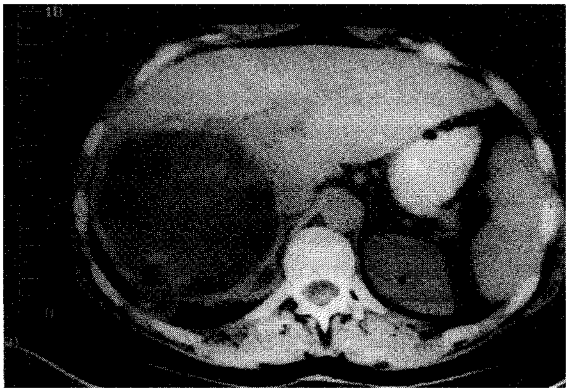

FIGURE 1 CT film showing 2 satellite cysts. Daughter cysts are also seen, into the main cyst.

TABLE I Location of the hydatid cysts ($n = 50$)

| | | | Total |
|---|---|---|---|
| Liver | • one lobe | 34 | 42 |
| | • two lobes | 8 | |
| Other organs | Lungs | 4 | |
| | Spleen | 2 | |
| | Kidney | 1 | 8 |
| | Ovary | 1 | |

43 patients underwent laparotomy and 7 patients underwent a right thoracotomy due to the localization of the hydatid cysts in the superior-posterior surface of the right hepatic lobe.

In 34 patients we succeeded in performing a total capsectomy and we removed 51 hydatid cysts. In 16 patients a total capsectomy was not feasible because as soon as we separated the fibrous capsule from the liver parenchyma we realised there was a risk to damage main arterial and venous branches of the liver, such as the portal vein, the hepatic veins, the inferior vena cava or main biliary branches. In these 16 patients we performed a partial capsectomy, evacuation of the content and omentoplasty. Table II summarizes the operative findings and complication rate in the two groups. Of the 51 hydatid cysts, where a total capsectomy was performed, small extra capsular cysts measuring 5–15 mm were found during separation of the main cyst from the liver parenchyma in 15 cases (29,5%) (Fig. 2). In 7 of these 15 cases the existence of the extracapsular cysts was already known following the preoperative radiological investigation.

TABLE II Operative findings and complication rate in the two groups

| | Satellite cysts | | Complications |
|---|---|---|---|
| Total capsectomy | Radiological findings | Operative findings | |
| $n = 34$ patients | 7 | 15 | 19% |
| Partial capsectomy | | | |
| $n = 16$ patients | 3 | none detected | 50% |

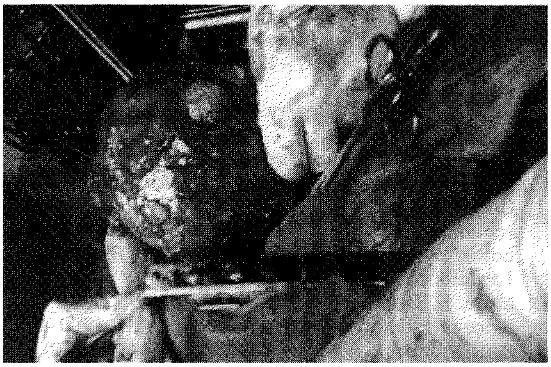

FIGURE 2 Operative picture of a big echinococcal cyst of the right lobe of the liver removed by cystopericystectomy through a right thoracotomy. Satellite cysts are clearly seen on the wall of the main cyst.

Complications in the group of 34 patients who underwent total capsectomy appeared in 7 patients (19%). Four patients developed haematoma and three patients had a bile leak. In the group of 16 patients who underwent partial capsectomy the complications were 8 (50%). The mean postoperative hospital stay for the group submitted to total capsectomy was 9 days. The mean postoperative hospital stay of the other group was 17 days. All patients were discharged from the hospital doing well. In the follow up period of 11 months to just over 5 years, one patient was re-operated for a hydatid cyst $3\frac{1}{2}$ years after the initial operation. He was the only case in the first group where the cyst was opened during surgery. Two patients in the group of 16 patients with partial capsectomy presented recurrence of hydatidosis.

### DISCUSSION

The existence of extracapsular hydatid cysts in the outer surface of the fibrous reactive capsule has been observed by surgeons during operative procedures of total or extended capsectomy. It is possible that this condition is responsible for a number of recurrences of the disease, when other factors are not incriminated. The mechanism of creation of these small extracapsular cysts is considered to be small ruptures of the capsule, as is the case of rupture into the biliary tract [7]. A retrospective study of the records of the Radiology Department of the Medical School, University of Athens (Aretaeion Hospital) for 5 years had been carried out by the authors [6, 7]. One hundred and eighty five cases of liver echinococcus glanulosus studied by computerized tomography were investigated. The material which was considered, concerned patients from many other hospitals as well as patients from our hospital. These cases were meticulously studied for the presence of small extracapsular cysts, outside the fibrous reactive capsule, separated or not from the main cystic cavity and they were found to be present in 30 cases, that is 16% [6, 7]. These cysts may "lie" on the fibrous capsule, they may be separated from the capsule, or they may protrude into the wall. We believed that the term "satellite" cysts is appropriate. Although their clinical significance has not been investigated they might be incriminated for recurrence of the disease. The presence of "satellite" cysts detected preoperatively by radiological studies was around 16% but the operative findings suggest that the real incidence is almost double, despite reviewing the relevant X-rays. This may be due to the small size or to the location of small cysts adjacent to the fibrous wall.

Total capsectomy, as a method of radical management of liver hydatidosis has been suggested because it avoids the spreading of the parasite intra-operatively, a known cause of recurrence which is estimated at 10–20% internationally [8–12]. In addition total capsectomy significantly reduces the problems which are associated with the remaining cavity of the hydatid cysts (abscess, protracted bile leak, long stay in hospital), which has been shown to be as high as 25–30% with other operative procedures [13, 14]. Only with total capsectomy are extracapsular cysts removed after a proper selection of patients [17–19]. It is noteworthy that we had a higher complication rate in the group of patients which underwent a partial capsectomy probably because this group presented the more difficult operative conditions. As hydatidosis tends to disappear perhaps only multicentre studies on a large number of patients and long follow up periods may give a more accurate number of the real incidence and the importance of extracapsular hydatid cysts.

### References

[1] Morris, D. and Richards, K. (1992). Hydatid Disease. Current Medical and Surgical Management. U.K.: Butterworth-Heinemann.

[2] Milicevic, M. (1994). Hydatid Disease. In: *Surgery of the Liver and Biliary Tract,* Edited by Blumgart, L. H., **79**, 1121–1150. Edinburgh, London, Madrid, Melbourne, New York and Tokyo: Churchill Livingstone.

[3] Morel, R., Robert, J. and Rohner, A. (1988). Surgical treatment of hydatid disease of the liver: A survey of 69 patients. *Surgery,* **104**, 859–62.

[4] Elhamel, A. (1990). Pericystectomy for the treatment of hepatic hydatid cysts. *Surgery,* **107**, 316–20.

[5] Moreno Gonzalez, E., Rico Selas, P., Martinez, Bercedo, Garcia Garcia, I., Palma Carazo, F., Hidalgo Pascual, M. (1991). Results of Surgical Treatment of Hepatic Hydatidosis: Current Therapeutic Modifications. *World J. Surg.,* **15**, 254–263.

[6] Voros, D., Kalovidouris, A., Gouliamos, A., Anastassopoulou, A. and Pissiotis, C. (1990). Investigation of exogenous cysts in hepatic echinococcal disease by computed tomography. Published summary In: *Surgical Research Communications,* **8**(1), 57.

[7] Kalovidouris, A., Voros, D., Gouliamos, A., Blachos, L. and Papavassiliou, C. (1992). Extracapsular (satellite) hydatid cysts. *Gastrointest. Radiol.,* **17**, 353–356.

[8] Kourias, B. (1974). Hydatid disease of the liver and other viscera. In: *Abdominal Operations,* Edited by Maingot, R., **2**, 1271–1292. New York: Appleton-Century Crofts.

[9] Saidi, F. (1976). Surgery of hydatid disease. W. B. Saunders Co.

[10] Pissiotis, C., Wander, J. and Condon, R. (1972). Surgical treatment of hydatid disease. *Arch. Surg.,* **104**, 454–459.

[11] Golematis, B. and Delikaris, P. (1984). Treatment of echinococcal cyst of the liver. In: *Mastery of Surgery,* Edited by Nyhus, L. and Baker, R., I:78, 633–41. Boston Toronto: Little, Brown and Company.

[12] Little, J., Hollands, M. and Ekberg, H. (1988). Recurrence of hydatid disease. *World J. Surg.,* **12**, 700–704.

[13] Papadimitriou, J. and Mandrekas, A. (1970). The surgical treatment of hydatid disease of the liver. *Br. J. Surg.,* **57**, 431–433.

[14] Demicri, S., Eraslan, S., Anadol, E. and Bozatli, L. (1989). Comparison of the results of different surgical techniques in the management of hydatid cysts of the liver. *World J. Surg.,* **13**, 88–91.

[15] Belli, Lino, del Favero, Ernesto, Marni, Antonio, Romani, Federico (1983). Resection *versus* pericystectomy in the treatment of hydatidosis of the liver. *Am. J. Surg.,* **145**, 239–242.

[16] Kalovidouris, A., Gouliamos, A., Vlachos, L., Papadopoulos, A., Voros, D., Pentea, S. and Papavassiliou, C. (1994). MRI of abdominal hydatid disease. *Abdom. Imaging,* **19**, 489–494.

[17] Filice, C., Pirola, F., Brunetti, E., Dughetti, S., Strosselli, M. and Foglieni, C. S. (1990). A new therapeutic approach for hydatid liver cysts. *Gastroenterology,* **98**, 1366–1368.

[18] Akhan, O., Dincer, A., Gokoz, A., Sayek, I., Havlioglu, S., Abbasoglou, O., Eryilmaz, M., Besim, A. and Baris, I. (1993). Percutaneous treatment of abdominal hydatid cysts with hypertonic saline and alcohol: An experimental study in sheep. *Invest. Radiol.,* **28**, 121–127.

[19] Akhan, O., Ozmen, M. N., Dincer, A., Sayek, I. and Gocmen, A. (1996). Liver hydatid disease: long-term results of percutaneous treatment. *Radiology,* **198**, 259–264.

